# Clinical translation of choline and geranic acid deep eutectic solvent

**DOI:** 10.1002/btm2.10191

**Published:** 2020-10-31

**Authors:** Justin Ko, Abhirup Mandal, Sunil Dhawan, Marina Shevachman, Samir Mitragotri, Nitin Joshi

**Affiliations:** ^1^ Department of Dermatology Stanford University School of Medicine Redwood City California USA; ^2^ CAGE Bio, Inc. San Carlos California USA; ^3^ Center for Dermatology Clinical Research, Inc. Fremont California USA; ^4^ John A. Paulson School of Engineering and Applied Sciences Harvard University Cambridge Massachusetts USA; ^5^ Wyss Institute of Biologically Inspired Engineering at Harvard University Boston Massachusetts USA

**Keywords:** CAGE, dermatology, ionic liquids, rosacea, topical, translation

## Abstract

Choline geranate deep eutectic solvent/ionic liquid (CAGE) has shown several desirable therapeutic properties including antimicrobial activity and ability to deliver drugs transdermally in research laboratories. Here, we describe the first report of clinical translation of CAGE from the lab into the clinic for the treatment of rosacea, a common chronic inflammatory skin disorder that affects the face. We describe the seven steps of clinical translation including (a) scale‐up, (b) characterization, (c) stability analysis, (d) mechanism of action, (e) dose determination, (f) GLP toxicity study, and (g) human clinical study. We describe the challenges and outcomes in these steps, especially those that uniquely arise from the deep eutectic nature of CAGE. Our translational efforts led to a 12‐week open‐label phase 1b cosmetic study with CAGE_1:2_ gel (CGB400) in mild–moderate facial rosacea in 26 patients where CGB400 exhibited a marked reduction in the number of inflammatory lesions. These results demonstrate the therapeutic potential of CGB400 for treating rosacea as well as it provides insights into the translational journey of deep eutectic solvents, in particular CAGE, for dermatological applications.

## 1 INTRODUCTION

Rosacea is a common chronic inflammatory skin disorder affecting 415 million people worldwide with highest prevalence in Caucasian adults (>10%) and clinical manifestations include facial erythema, papulopustular lesions, phymatous changes, facial telangiectasias, intermittent flares of acute vasodilation (flushing).^1^, ^2^ It is a phenotypically heterogenous disease with varying range of clinical manifestations and symptoms that makes the diagnosis and treatment challenging.^3^, ^4^ While the underlying causes of rosacea remain poorly defined, therapies that target multiple contributing factors may offer advantages over current therapies.^5^, ^6^

A few topical products have been approved by the FDA for the treatment of rosacea including Mirvaso (0.33% brimonidine) topical gel^7^ and Rhofade (1% oxymetazoline hydrochloride)^8^ for persistent (nontransient) facial erythema of rosacea in adults. Additional products include Soolantra (1% ivermectin cream),^9^ Finacea (15% azelaic acid foam),^10^ and Metrogel (1% metronidazole gel)^11^ which have gained specific investigative interests for crucial role in modulating immune responses and selective antiparasitic activity in rosacea pathophysiology.^12^, ^13^ Mirvaso and Rhofade are vasoconstrictors and address only transient redness. They have limited efficacy against persistent redness, inflammatory papules or pustules. Soolantra, Finacea, and Metrogel have shown antimicrobial and anti‐inflammatory effects in reducing the inflammatory papules and pustules associated with the disease; however, their effect on persistent facial erythema has been severely limited.^14^ They are also limited by treatment cost, poor dermal permeation, and have certain regional restrictions of manufacturing.^15^ Hence, the development of therapeutics that address these challenges can significantly improve the treatment and care of rosacea.^16^, ^17^

Recently, an ionic liquid/deep eutectic solvent comprised of choline and geranic acid (CAGE) has been shown to exhibit characteristics that make it a potential candidate for effective treatment of rosacea. Specifically, prior in vitro studies have shown that CAGE exhibits excellent antimicrobial activity against a broad variety of pathogens^18^, ^19^ including *Staphylococcus epidermidis* which could be associated with pustules of rosacea.^20^ Further, CAGE has been shown to exhibit deep penetration into the skin, thus suggesting its ability to treat pathogens residing deep into the skin.^21^ Building on this scientific foundation, here we demonstrate successful clinical translation of this innovative technology into a formulation referred to as CGB400, comprising 40% w/w CAGE_1:2_ (choline:geranic acid ratio of 1:2) gel formulated in common pharmaceutical excipients. Conversion of academic discoveries into clinical products is often limited by numerous hurdles of practical as well as fundamental origins. Specifically, academic innovations which are not usually constrained by issues of scalability, long‐term stability and human safety may not be compatible with the requirements of a useful clinical product. Thus, many scientific discoveries, in spite of their significant potential, fail to reach the clinic. Here, we describe the journey of CAGE, which was first reported as an academic scientific discovery in 2014,^19^ through the “seven steps of translation” including scale‐up, characterization, stability, mechanism of action, dosing, preclinical toxicology in minipigs and finally, human volunteer studies on safety and efficacy in treating rosacea. Collectively, these studies report the first example of clinical translation of an ionic liquid‐based approach for the treatment of dermatological conditions. We also share the learnings through these seven steps of translation that may be helpful for other translational activities in the field.

## 2 RESULTS

### 2.1 Translational step 1: CGB400 synthesis and scale‐up

**FIGURE 1:**
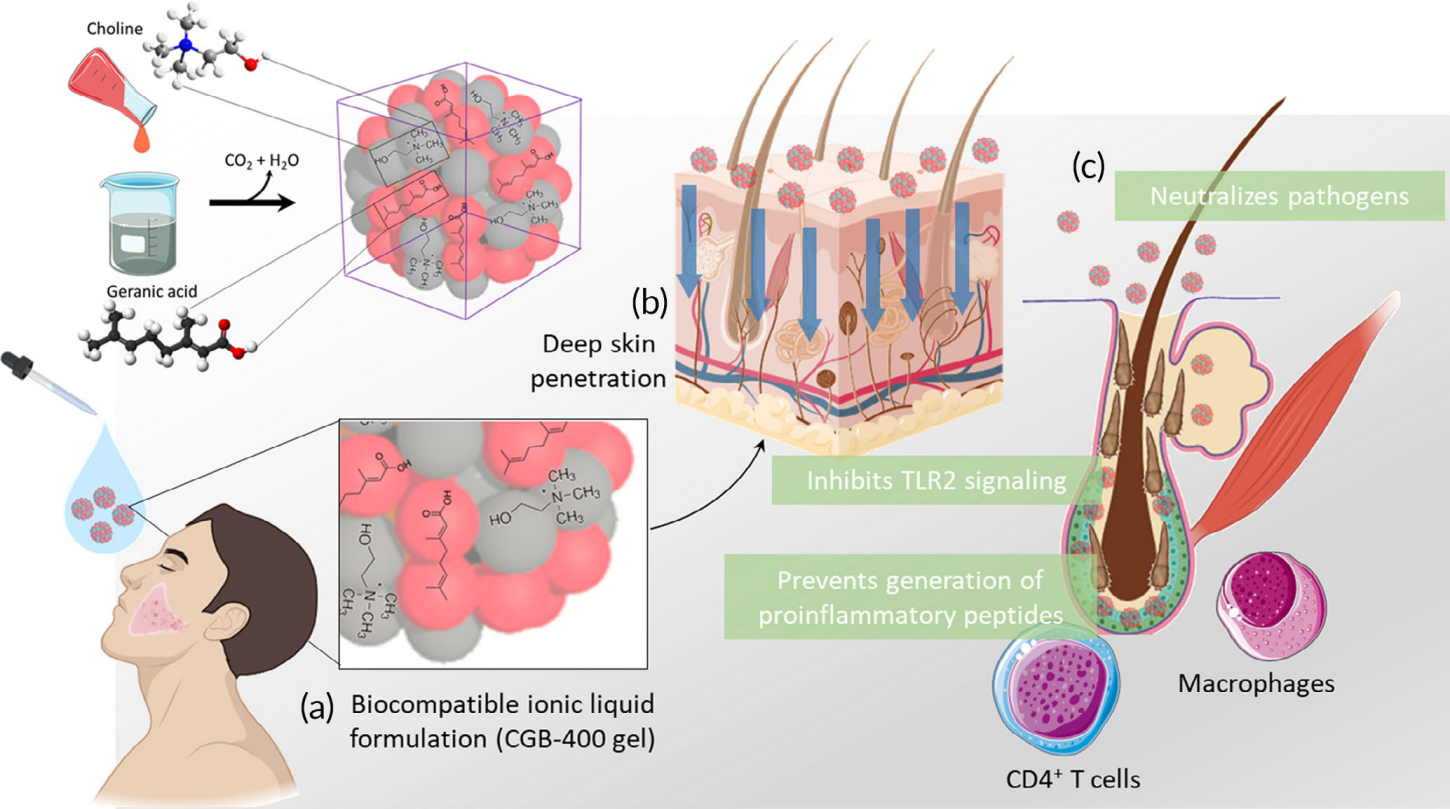
Schematic illustration of mode of action of choline and geranic acid (CAGE) for rosacea. (a) Synthetic scheme of CAGE/CGB400 gel, deep eutectic mixture. (b) CGB400 upon topical application, extracts lipid from the lipid bilayer and penetrates deep into the skin layers. (c) CAGE neutralizes pathogens, modulates TLR2 signaling, and inhibits KLK5 and thus generation of pro‐inflammatory peptides

Previous reports of synthesis of CAGE_1__:2_ was performed at a laboratory scale, with typical batch sizes of several grams.^19^ While this is often adequate for research level use, this scale is not adequate for human testing. Scale‐up of materials is often a complex process since some of the key functional aspects of the synthesis procedure may not scale proportionately. The active ingredient in CGB400, CAGE_1__:2_, was synthesized in a commercial reactor at a kilogram scale using the same process as that used at a smaller scale. Specifically, CAGE_1__:2_ was synthesized by a one‐step salt metathesis reaction between choline bicarbonate and geranic acid (Figure 1(a)). No specific issues were observed during scale‐up. CAGE_1__:2_ is hygroscopic in nature and is miscible with water. During salt metathesis, evolution of CO_2_ gas was monitored to assess the completion of the reaction. The resulting deep eutectic ionic liquid possessed both fluidity and transparency at room temperature with approximately 13% water content. The solution is a clear, colorless to yellow colored viscous liquid with a pH ~8.5, conductivity ~1.3 mS/cm, and a characteristic odor. Reaction duration and process parameters were optimized to improve manufacturing scale‐up with the end product quality, defined by the water content, pH, conductivity and appearance. The initial scaled formulation (>3 kg) was designed to produce CAGE_1__:2_ with the desired homogeneity for nonclinical studies. CGB400 gel was manufactured by mixing the CAGE_1__:2_ solution with water, propylene glycol as co‐solvent, d‐limonene as a fragrance, and hydroxypropyl cellulose as a gelling agent. The inactive ingredients used in the CGB400 gel are inert in nature and were chosen to minimize dissociation of CAGE_1__:2_ or have minimal effect on the CAGE_1__:2_ structure.

Then, 5 kg batches of CGB400 gel were manufactured at an experienced contract manufacturing organization in California for the human studies. Preservatives are often needed to protect against the growth of microbes in the formulation. However, since CGB400 gel itself possessed antimicrobial properties due to the inherent antimicrobial activity of CAGE_1:2_, the formulation did not require additional preservation; keeping it paraben‐free. All excipients used were USP/NF grade, except the fragrance agent which is a food‐grade material.

### 2.2 Translational step 2: Characterization of CAGE_1:2_

Adequate characterization steps form the foundation of product development. For small molecules drugs, methods such as chromatography and NMR, among others, have been well‐established over the years. As newer drugs such as biologics emerged on the landscape, the field of drug development has collectively rushed to develop the necessary methods for such products. This is one of the key challenges for deep eutectic solvents/ionic liquids. As an emerging category of actives, methods for characterizing these materials as drug products are in infancy. A key challenge is that the standard chemical methods are not adequate since no new covalent bond is formed during the salt metathesis between choline and geranic acid, thus making over reliance on conventional methods characterization challenging. CAGE_1:2_ was chemically characterized using NMR and HPLC analyses (Figure S1). 1H NMR spectra of CAGE_1:2_ indicated notable differences in the chemical shifts compared to the individual components. These chemical shifts are pronounced for the protons localized adjacent to the quaternary nitrogen atoms indicating the strong anion effect of the anion, geranic acid. HPLC did not exhibit a clear signature of CAGE compared to its individual components. Specifically, the retention times (RT) of major peaks in the chromatogram of CAGE_1:2_ corresponded to that obtained in choline and geranic acid standard solutions. Both methods confirmed the presence of choline and geranic acid in CAGE_1:2_. However, in the absence of a covalent bond between them, these methods were deemed necessary but not sufficient.

The physical and chemical properties of CAGE_1:2_ were additionally evaluated using modulated differential scanning calorimetry (MDSC) and thermal gravimetric analysis (TGA) and Fourier transform infrared spectroscopy (FTIR). MDSC indicated a glass transition temperature (Tg) around −68°C (Figure S2). This is illustrated in both the reversible heat flow and reversible heat capacity curves in both heating and cooling. It is likely that the liquid CAGE_1:2_ formulation solidifies into an amorphous solid. The small exotherm during cooldown at −80°C (only apparent at 1°C/min) might be associated with an ordering event to an extent, like partial crystallization (Figure S3). From the TGA data, it is evident that CAGE_1:2_ lost approximately 4% weight at approx. 140°C, likely water. The sample completely evaporated or decomposed starting around 165°C. No hysteresis was observed in the sorption/desorption isotherm of the gravimetric vapor sorption which indicated that CAGE_1:2_ is hygroscopic (Figure S2). The salt metathesis reaction was also monitored via FTIR spectroscopy (Figure S4). The broad peak at 3300 cm^−1^ most likely corresponds to the combination of choline —OH bond and carboxylic acid in geranic acid as is evident in CAGE_1:2_. Additionally, the presence of peaks at 1690 and 2968 cm^−1^ due to C=O and OH stretch of carboxylic acid in choline geranate confirms the presence of individual components of the ionic liquid.

In case of CAGE_1:2_, it has been observed that partial dissociation of the anion and cation occurs in the presence of water. We anticipate that a dynamic equilibrium exists between CAGE_1:2_, individual components and water. At concentrations below 20%, CAGE_1:2_ transitions from a bulk deep eutectic/ionic liquid structure into vesicles and transitions to microemulsion at much lower concentrations.^22^

### 2.3 Translational step 3: Stability under forced degradation conditions

Long‐term stability of the drug product is a key requirement. It should be noted that the components of CAGE_1:2_, that is, choline and geranic acid are GRAS materials with well‐established safety profiles. No specific toxic impurities in either of these materials have been reported. Impurities in CAGE_1:2_ and CGB400 were quantified using validated HPLC methods. The limit for each impurity obtained at specific RTs was monitored at lot release and during the stability studies. Stability of CGB400 was tested under forced degradation conditions following standard protocols. CGB400 formulation, upon exposure to heat or UV‐A stress demonstrated minimal change in stability with a 0.6 and 0.4% loss of choline peaks, respectively. A few new unknown peaks appeared in the chromatographic profiles of vehicle as well as the sample after heat and UV‐A stress, but they did not interfere with the determination of choline. Similarly, for geranic acid the heat‐, UV‐, acid‐, base‐, or peroxide‐stressed vehicle contained no peaks that interfered with the determination of geranic acid. The heat‐, UV‐, acid‐, base‐, or peroxide‐stressed CGB400 gel showed a 20.0, 2.0, 0.3, 0.0, and 3.6% loss of geranic acid, respectively.

Solution stability of CGB400 gel was also tested. The stored samples and standards met the 95.0–105.0% recovery of initial concentration of choline and 97.0–103.0% recovery of initial concentration of geranic acid per the validation protocol criteria. The chemical and physical stability of CGB400 gel was evaluated over a 12‐month period. The CGB400 gel was found to be stable in accelerated (40C/75% RH), intermediate conditions (30C/65% RH), and long‐term storage conditions (25C/60%RH) with relatively low increase in impurities level (total impurities level was increased from 0.42 to 2.5%). Moreover, microbial limit that was tested at every time point of the stability study was found to be in the acceptance range for topical gel products suggesting that CGB400 gel is well preserved for a period of at least 12 months. There was a slight increase in the pH (~0.4) and change in viscosity (~1000 cP) of the CGB400 gel at intermediate and RT conditions over a 12 months period. It is noteworthy that the change in viscosity observed over 12 months is not unusual for hydroxyethyl cellulose gels without a stabilizer. Inclusion of an antioxidant can potentially address this limitation. Additionally, no significant variation in the physical appearance, color, and odor was observed. The minor changes in the visual appearance of the gel continued to meet specifications. We anticipate that the addition of an antioxidant would address the slight color change and viscosity drop of CGB400. The packaging for the GLP and cosmetic studies was performed in amber glass bottles.

### 2.4 Translational step 4: Mechanism of action of CAGE_1:2_

**FIGURE 2:**
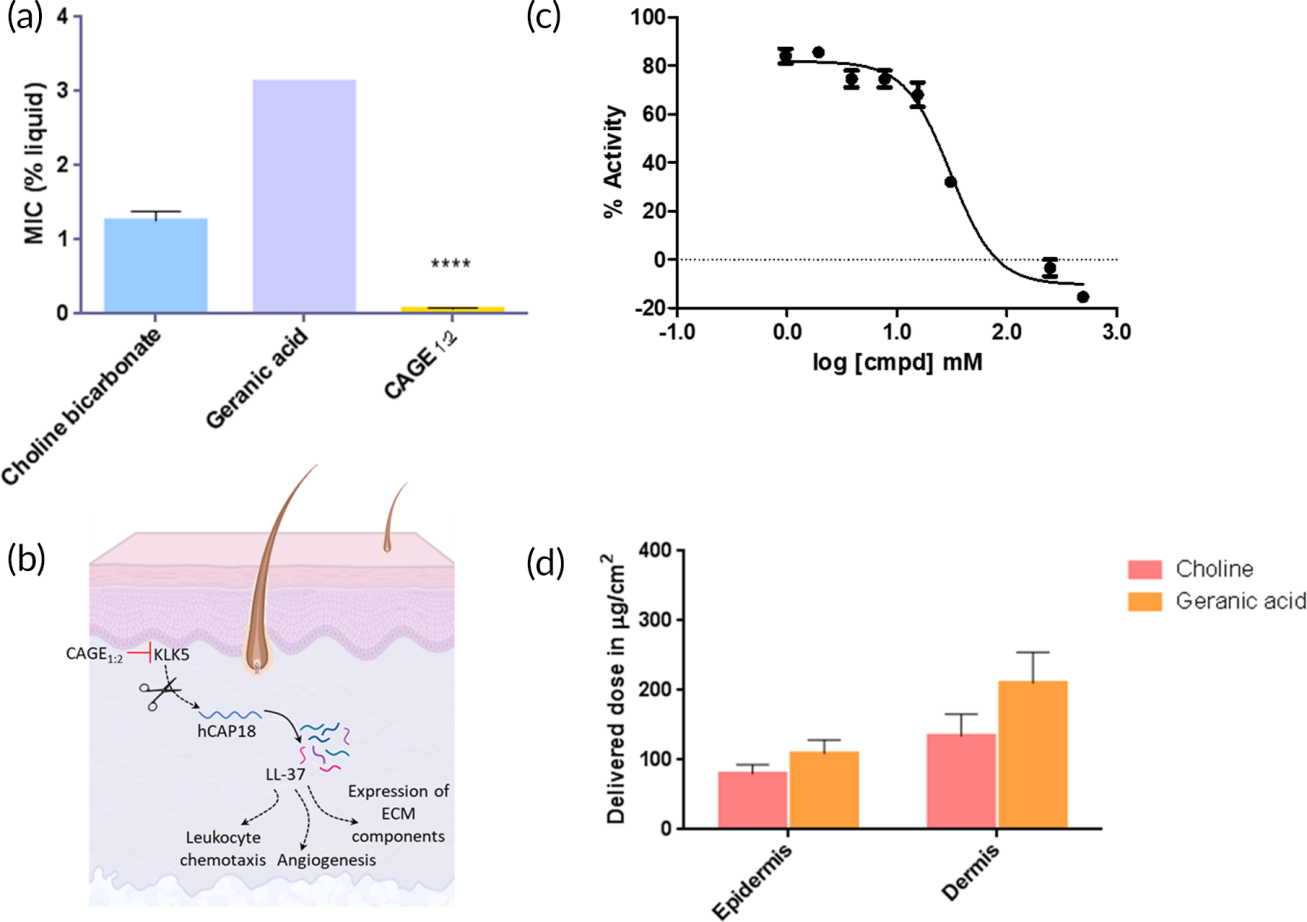
Therapeutic activity and skin permeation of CGB400. (a) Broth microdilution minimum inhibitory concentrations (MICs) for choline bicarbonate, geranic acid, and CAGE_1:2_ (****p < 0.0001); (b) schematic of KLK5 inhibition pathway (c) IC50 curve of CAGE; and (d) comparison of in vitro skin quantification of choline and geranic acid in human cadaver skin following CGB400 gel application. Data are averages ± *SEM*, statistics by one‐way ANOVA with Tukey HSD posttest. ****p < 0.0001

While not an essential element of the translational path, an understanding of the mechanism of action can provide the knowledge that enables making rational choices when multiple dosing or formulation options are feasible. The biological origin of rosacea is relatively less understood. However, studies have clearly demonstrated the role of resident bacteria and the activity of kallikrein 5 enzyme (KLK5).^23^, ^24^ Previous studies have demonstrated excellent antiparasitic efficacy of CAGE_1:2_ against a breadth of bacterial, fungal and viral species.^18^ The ability of CAGE_1:2_ to inhibit *P*. *acnes* as a model gram positive bacterium was tested using the CLSI standard broth microdilution methodology (BMD) (described in SI). CAGE_1:2_ demonstrated high antibacterial activity against *P*. *acnes* isolates (Figure 2(a)). The minimum inhibitory concentration (MIC) of CAGE_1:2_ ranged from 0.025 to 0.2% of neat CAGE_1:2_ (or approximately 57 μM–0.45 mM of CAGE_1:2_). These results demonstrate the potential of CGB400 as an effective antimicrobial agent to treat underlying pathogenic origins in rosacea. Of particular note, the MIC of CAGE_1:2_ was significantly lower than that of its individual components choline bicarbonate and geranic acid (*p* < 0.0001), thus indicating that the effect of CAGE_1:2_ on microbial activity is not an additive effect of the individual antimicrobial activity of choline or geranic acid, but clearly an effect that is attributed to the composition of CAGE_1:2_. Importantly, at lower concentrations, some portion of CAGE_1:2_ maintains the properties of a deep eutectic/ionic liquid and exhibits far superior antimicrobial activity compared to the individual components at the same concentrations.^25^

CAGE_1:2_ also inhibited KLK5. Effect of CAGE_1:2_ on hKLK5 was studied and compared to aprotinin as a positive control. IC_50_ values of CAGE_1:2_ and aprotinin were found to be 30.84 mM and 0.92 μM, respectively, indicating that CAGE_1:2_ is effective in inhibiting hKLK5 activity, although its potency is lower than that of aprotinin (Figure 2(b) and (c), Figure S5, and Table S1).

### 2.5 Translational step 5: Determination of dosing of CGB400

Mechanistic studies pointed out effective concentrations of <0.45 mM for antibacterial activity and 30.84 mM for KLK5 inhibition. However, these concentrations must be achieved deeper into the skin including the epidermis and the dermis. Towards that goal, the permeation of choline and geranic acid from CGB400 gel into skin was evaluated in vitro using human cadaver skin. Since no covalent bond is formed between choline and geranic acid during the synthesis of CAGE_1:2_, choline and geranic acid were separately detected in the skin. CGB400 gel exhibited excellent skin penetration generating high concentrations of its constituents, choline and geranic acid in the skin. The delivered doses of choline in the epidermis and dermis were 80.2 ± 13.0 and 134.6 ± 30.9 μg/cm^2^, respectively, and the delivered doses of geranic acid were 109.3 ± 19.5 and 210.7 ± 44.3 μg/cm^2^ in the epidermis and dermis, respectively (Figure 2(d)). The molar ratios of choline to geranic acid in the epidermis were ~1.18 and that in the dermis was ~1.03 at the end of 24 h. A molar ratio of choline:geranic acid of 1.18 in the epidermis, though higher than that in the formulation (0.5), is indicative of the association of choline and geranic acid during the skin penetration of CAGE_1:2_. Specifically, choline and geranic acid have substantially different lipophilicities (LogP of geranic acid, 2.8 and LogP of choline, −3.7). Accordingly, their individual skin permeabilities are expected to be dramatically different. Hence, the closeness of the choline:geranic acid molar ratio in the skin to that in the applied formulation suggests the existence of interactions between choline and geranic acid during skin penetration. Penetration of CAGE into skin had a quick onset. Specifically, the fluxes of choline and geranic acid in the epidermis after 8 h of formulation placement were 3.48 ± 0.55 and 27.54 ± 1.94 μg/cm^2^/h, respectively. An epidermal choline dose of 80.2 ± 13.0 μg/cm^2^ leads to an estimated epidermal CAGE concentration of ~76 mM assuming an epidermal thickness of 100 μm, a number in the target range of therapeutic activity.

### 2.6 Translational step 6: GLP dermal toxicity in minipigs

Minipigs are considered an excellent model for studying toxicity of topical skin formulations.^26^, ^27^ GLP toxicity studies were performed in Göttingen minipigs by applying the CGB400 gel once a day for 91 consecutive days. Total of 1 ml CGB400 was applied over 10% of the total body surface area of the animal. All animals survived to scheduled study termination and there were no macroscopic findings. No clinical signs, changes in body weight, food consumption, ocular, electrocardiography, hematology, coagulation, clinical chemistry, urine analysis, and organ weights parameters were observed. Dermal changes including edema, erythema, fissuring, and focal red spots were observed in CGB400‐treated animals and completely disappeared by the end of 14‐day recovery period. Overall, CGB400 was well tolerated in minipigs.

**FIGURE 3:**
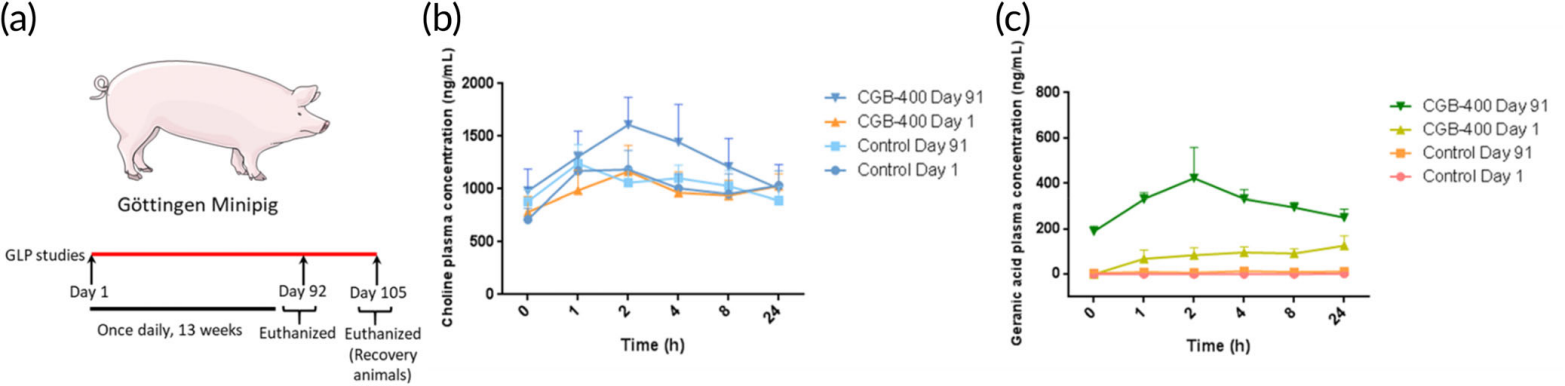
GLP dermal toxicity of CGB400 in minipigs. (a) Dosing regimen in Gottingen minipig; plasma concentrations of choline (b) and geranic acid (c) on Days 1 and 91 in comparison to control following dermal administration

Toxicokinetic (TK) parameters were determined from plasma concentration of choline and geranic acid. The mean ± SE *C*_max_ values for choline on Day 1 for male and female were 609 ± 169, 459 ± 106; and on Day 91 were 783 ± 196, 1080 ± 492 ng/ml, respectively. Besides, the AUC0‐Tlast on Day 1 for male and female were 4610 ± 3570, 5350 ± 1680; and on Day 91 were 6750 ± 2650, 3110 ± 776 h × ng/ml, respectively. Over the 91‐day treatment period, *C*_max_ and AUC accumulation ratios of choline (Day 91/Day 1) were 1.3 and 1.5, respectively, in males and 2.4 and 0.6 in females, demonstrating accumulation to a certain extent when CGB400 gel was applied by dermal administration to Göttingen minipigs daily for 13 consecutive weeks. It is worth noting that the choline levels measured are not exclusive to the exogenous choline given to the animals through the CGB400 gel administration, as they were levels of choline ingested through the feeding as well as endogenous levels of choline. In fact, the choline levels for the control group (treated with a vehicle containing ~90% water along with propylene glycol, a fragrance, and a gelling agent) are indicative of the baseline levels of choline in plasma. In contrast, the mean *C*_max_ values for geranic acid on Day 1 for male and female were 197 ± 64.1, 68.5 ± 24.3; and on Day 91 were 373 ± 44.2, 573 ± 254 ng/ml, respectively. In addition, the AUC0‐Tlast on Day 1 for male and female were 3560 ± 938, 1260 ± 459; and on Day 91 were 7050 ± 826, 6950 ± 1390 h × ng/ml, respectively. *C*_max_ and AUC accumulation ratios of geranic acid (Day 91/Day 1) were 1.9 and 2.0, respectively, in males and 8.4 and 5.5 in females, suggesting consequential accumulation of geranic acid. The plasma concentrations of choline and geranic acid on Days 1 and 91 in comparison to control following dermal administration have been depicted in Figure 3.

TK studies in pigs also included two additional doses, one at 1.5× of CGB400 (i.e., 60% CAGE_1:2_) and one at about twice that of CGB400 (i.e., 87% CAGE_1:2_, which is pure CAGE_1:2_ which naturally absorbs water to form a 87% mixture). Generally, similar results were obtained at these doses and the plasma concentrations of geranic acid generally scaled with the dose (Figure S6).

### 2.7 Translational step 7: Clinical evaluation in human volunteers

**FIGURE 4:**
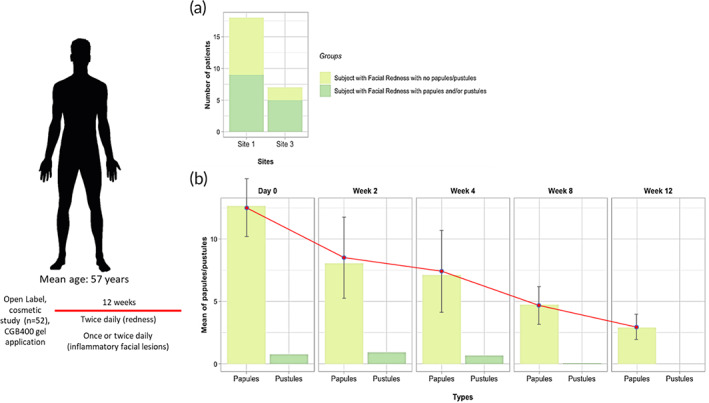
Efficacy in patient subjects. Overview of (a) number of patients in different groups at the two sites and (b) mean of papules/pustules in patients with “redness with bumps/blemishes”

In an IRB‐approved 12‐week open label cosmetic study for rosacea, subjects with a mean baseline lesion count (papules and pustules) of 13.4 ± 6.7 lesions were presented. Three sites were used for the study and the median age of subjects was 57 years (Figure 4(a)). After 2 weeks of therapy with CGB400 gel, the mean papules and pustules count was reduced by 34.6% (*p* = 0.0018) with further reductions in lesion counts being observed at each subsequent study visit. The greatest reduction in lesion counts was seen at Week 12 (Visit 5) where a mean drop from baseline of 71.9% (*p* < 0.0001) was observed (Figure 4(b)).

It is worth noting that the CGB‐400 gel dose applied daily on the minipig's skin was 1 g, where each gram of gel contained 0.4 g of CAGE. At the highest concentration (87%), the applied dose of CAGE_1:2_ was 0.87 g per day, which is equivalent to 69.3 mg/kg/day, assuming 12.55 kg as an average weight of a minipig. For the studies in human volunteers, ~0.2 g of a drug product was applied twice a day, that is, 0.08 g of CAGE_1:2_ per application or 2.3 mg/kg/day for a 70 kg adult. As previously established, considering minipig as the most predictive animal model for human percutaneous absorption, the target human dose is ~×30 lower than the highest dose evaluated in the repeat dose toxicity study in minipigs and thus justifies the translational potential of CGB400 gel.^28^, ^29^, ^30^

In addition, investigator global assessment (IGA) responder rates (i.e., ≥1‐point decrease from baseline) as well as the proportion of subjects with IGA scores of “clear” or “almost clear” were assessed. Both IGA measures demonstrated accumulated effects throughout the study. Therapeutic response was noted as early as Visit 2 (Week 2), where 44.4% [25.5, 64.7] of subjects were deemed to be IGA “responders,” and 29.6% [13.8, 50.2] of subjects were clear/almost clear. Response continued to strengthen out to Visit 5 (Week 12) where 82.6% [61.2, 95.1] of subject were deemed to be IGA “responders,” and 69.6% [47.1, 86.8] of subjects were clear/almost clear.

Furthermore, IGA‐redness (IGAR) responder rates (i.e., ≥1‐point decrease from baseline) as well as the proportion of subjects with IGAR scores of “clear” or “almost clear” were assessed. Both IGAR measures demonstrated accumulated effects throughout the study. IGA captured investigators assessment of inflammatory lesions on the face whereas IGAR specifically captured facial redness, independent of lesions. Therapeutic response was noted as early as Visit 2 (Week 2), where 22.2% [8.6, 42.3] of subjects were deemed to be IGAR “responders.” However, only 3.7% [0.1, 19.0] of subjects were clear/almost clear. Response continued to strengthen out to Visit 5 (Week 12) where 69.6% [47.1, 86.8] of subject were deemed to be IGAR “responders,” and 47.8% [26.8, 69.4] of subjects were clear/almost clear. Likewise, in patients without bumps/blemishes, 50.0% [27.2, 72.8] and 52.9% [27.8, 77.0] of subjects were deemed to be IGAR “responders” at Visit 2 (Week 2) and Visit 3 (Week 4), respectively. Effectiveness evaluations based on PGA, IGA, and IGAR in subjects with facial redness with/without bumps/blemishes (Figures S7 and S8).

Patient global assessment (PGA) responder rates (i.e., ≥1‐point decrease from baseline) were also assessed. After only 2 weeks of treatment, the majority of subjects (i.e., 51.9% [32.0, 71.3]) were already PGA responders. Response continued to strengthen out to Visit 5 (Week 12) where 73.9% [51.6, 89.8] of subjects were deemed to be PGA “responders.” Likewise, for patients without bumps/blemishes, after 2 weeks of treatment, 6 (31.6% [12.6, 56.6]) subjects were PGA responders and the response strengthened out to Visit 3 (Week 4) where 50.0% [24.7, 75.4] of subjects were deemed to be PGA “responders.”

Safety assessment indicated 8 (29.6% [13.8, 50.2]) subjects with bumps/blemishes experienced a total of 11 treatment‐related adverse events (TRAEs). There were no SAEs reported in the study. Moreover, six (24.0% [9.4, 45.1]) subjects experienced a total of nine treatment‐emergent adverse events, and five (20.0% [6.8, 40.7]) subjects experienced a total of eight TRAEs. One SAE (unrelated to study treatment) was reported by one subject (heart valve replacement) in patients without bumps/blemishes (Table S2).

## 3 DISCUSSION

Here, we report clinical translation of a novel ionic liquid/deep eutectic solvent CAGE for the treatment of rosacea. CAGE has been previously reported as an engineered ionic liquid/deep eutectic solvent that possesses excellent skin permeation, antimicrobial activity and the ability to enhance drug delivery into skin.^31^, ^32^ While extensive in vitro and in vivo assessment of CAGE has been reported in the literature,^22^, ^33^ its translation into preclinical development and clinical validation has not been reported.

CAGE_1:2_ was synthesized using a one‐step salt metathesis reaction. Process development approaches were leveraged to overcome the limitations of laboratory scale CAGE_1:2_ synthesis. Industrially relevant, commercial reactor and processes enabled monitoring of reaction duration, CO_2_ evolution and temperature for maintaining key quality attributes like pH, viscosity, color, odor, and conductivity. The staple synthetic steps further rendered the manufacturing scale‐up of CAGE_1:2_ and eventually CGB400 highly economical and straightforward. Furthermore, no additional preservatives were required in the final product, CGB400 topical gel owing to the inherent antimicrobial properties of CAGE_1:2_. Methods for characterizing the product were developed and used to assess the finished product as well as its stability. The formulation exhibited excellent stability under stressed storage conditions.

Mechanistically, CAGE_1:2_ appeared to target two key pathways know in rosacea. Specifically, higher densities of *S*. *epidermidis* have been thought to be responsible for inflammation associated with rosacea.^20^, ^34^ Further, activation of epidermal proteases, especially kallikrein (KLK‐5) is also associated with rosacea and may further contribute to the inflammatory process.^35^, ^36^ KLK‐5 is a protease responsible for synthesis of antimicrobial peptides and its overexpression in the skin increases the local concentrations of the antimicrobial peptide, thus leading to overactivation of the local innate immune response which in turn causes cutaneous inflammation, vasodilation, and vascular proliferation.^24^ Activation of reactive oxygen species and hydroxyl radicals in the skin may also be a part of the inflammatory response, suggesting the need for agents modulating wider mechanistic and therapeutic targets.^37^

CAGE_1:2_ exhibited efficacy against both targets. It exhibited strong activity against 10 different clinical isolates of *P*. *acnes*. Previous studies have shown that CAGE_1:2_ penetrates into bacterial membrane and induces it disruption.^19^ Upon arriving near the bacterial membrane, choline interacts with the negatively charged bacterial membrane, thus inducing the geranic acid tail to enter the bacterial membrane leading to disruption. The mechanism by which CAGE_1:2_ inhibits KLK serine protease family, KLK5, is unclear. IC_50_ of CAGE against KLK5 was found to be ~30 mM. in vitro skin permeation studies in human skin indicated that the epidermal and dermal concentration after 24 h far exceeded this value, thus confirming that the therapeutic concentration of CAGE_1:2_ can be achieved. Further studies should focus on evaluating the mechanisms by which CAGE_1:2_ impacts the broader involving the innate immune system.

In vitro diffusion cell studies indicated that CAGE_1:2_ exhibited quick and extensive permeation into the epidermis and dermis. About 9.5% of the applied CAGE_1:2_ dose penetrated into the dermis within 24 h, as determined by the amount of geranic acid recovered from the skin. Previous studies have established that CAGE_1:2_ contributes to lipid extraction from the stratum corneum (SC) lipid bilayers, which promotes skin's permeability to agents including CAGE itself. Simultaneous measurement of choline and geranic acid in the skin indicated that the ratio of choline:geranic acid in the epidermis was 1.18 after 24 h. This is a surprising finding given that the lipophilicities of geranic acid and choline, as measured by octanol–water partition coefficients, differ by seven orders of magnitude. This strongly suggests that choline and geranic acid maintain some interaction during their skin penetration. Furthermore, the higher concentration of choline in the skin and hence a higher molar ratio of choline:geranic acid (1.18) than that in the formulation (0.5) is likely due to the endogenous levels of choline.

GLP toxicity studies in minipigs indicated that long‐term topical application of CGB400 for 91 days was generally well tolerated with no mortality and no macroscopic changes. Some dermal changes were observed but disappeared completely following 14‐day recovery period which supports the safety profile of CGB400 gel. Furthermore, TK profile indicated no significant increase in the measured blood choline concentrations from the existing levels. This could potentially have originated from the fact that dietary choline and/or natural choline metabolism dominated the blood concentrations. In contrast, the levels of geranic acid increased in the blood in a dose responsive manner. No systemic effects or organ toxicity of increased geranic acid concentration were seen.

CGB400 was well tolerated by patients, with most local side effects being mild to moderate. In an open‐label 12‐week cosmetic study, CGB400 demonstrated significant reduction in lesion counts (papules and pustules) on just Visit 2 (2 weeks) with further reductions at subsequent study visits. At Visit 5 (Week 12), greatest reductions (~71%) in lesions were reported, confirming the efficacy of CGB400 gel in improving rosacea symptoms. IGA responder rates and scores clearly demonstrated a marked improvement in symptoms with ~70% of the subjects were deemed clear/almost clear. Likewise, IGAR scores strengthened until Week 12, with ~48% of clear/almost clear subjects. Furthermore, ~73% subjects were deemed PGA responders after 12‐week treatment. These findings confirm significant potential of CGB400 gel in the treatment of rosacea.

The studies reported here are a proof‐of‐concept (POC) assessment and demonstration of the translational path of a deep eutectic solvent/ionic liquid. Future studies should include comparators to assess the efficacy of CGB400 in direct comparison to current products. Further clinical studies should also include vehicle controlled randomized trials. While the studies reported here focus on rosacea, they provide guidance for many other potential products that use CAGE. In particular, the processes related to scale‐up, characterization, stability, and GLP‐toxicity could be common to many other products based on CAGE. Unlike small molecules and biologics where a number of successful examples have paved the path for translation of new molecules, ionic liquids are still infancy. Owing to their unique nature, they require new methods or considerations for synthesis and characterization as discussed here. These findings may provide a path for additional future translational studies for ionic liquid‐based products.

## 4 MATERIALS AND METHODS

### 4.1 One‐step lab scale formulation of CAGE_1:2_

Choline geranate (CAGE_1:2_) was synthesized as described previously. Briefly, the cation, choline bicarbonate and geranic acid, anion was mixed at a 1:2 ratio to prepare IL/deep eutectic mixture following salt metathesis reaction. Geranic acid (3.696 mol) was weighed in a stainless‐steel vessel equipped with a stirrer and placed into a water bath. Choline bicarbonate 80% solution (1.848 mol) was added dropwise to the vessel. The mixture was reacted for 8 h at 27°C. The water content of the resulting IL/deep eutectic mixture was measured using Karl Fischer titration as an in‐process parameter.

### 4.2 CAGE_1:2_ characterization

Thermal analysis (MDSC and TGA) and moisture sorption analysis (dynamic vapor sorption [DVS]) of CAGE_1:2_, eutectic mixture was carried out by CeutixLabs, San Diego. The method development for MDSC and TGA have been discussed in detail (Supplementary materials and methods). For the MDSC, sample was dried for 3 h at 60C on the TGA prior to running the DSC. The sample was quickly sealed (hermetically) after drying on the TGA which helped in removing most of the moisture and water associated thermal events (there may be a very low fraction of water left, that is, <0.5%). There were two parts to the MDSC analysis including run at 3°C/min followed by run at 1°C/min. The run at 1°C/min was better controlled over a wider range. It should be noted that the modulation signal gets a little distorted <‐80C during the cooling cycle, so does not read the fine details below −80C. The TGA was ran in Hi‐Res mode, that is, a dynamic (variable) heating rate where the heating rate is inversely proportional to the weight loss rate. On the Y‐2 axis of the TGA plot, the derivative of the temperature vs time was plotted. This was simply to illustrate the instantaneous heating rate (degrees/min) at any particular temperature during the run. This shows that the dynamic heating rate varied from nearly 0 (around 160°C—when the weight dropped off the cliff) to 50°C/min near the end of the run. DVS, using gravimetric moisture sorption (GVS) analysis was also conducted. Both TGA and GVS analyses have been considered since, TGA measures volatiles in % wet weight, whereas GVS measures water uptake with respect to dry weight. Additionally, FTIR analysis of choline, geranic acid and CAGE_1:2_ were carried out and compared using FTIR Spectrometer, PerkinElmer Spectrum One. Report builder, Rev 2.01 (software) was used for spectra analyses.

### 4.3 Antimicrobiological in vitro activity

CAGE_1:2_ and its individual components were tested for any antibacterial activity against selected organisms, using the CLSI standard BMD. Clinical isolates of *Propionibacterium acnes* (newly classified as *Cutibacterium acnes*) were employed in this study. Considerable precipitate was observed when each of the test components was added to the media at a 1:2 dilution. Therefore, preliminary concentration checks were performed to determine the highest concentrations of the liquid to be utilized.

### 4.4 hKLK5 enzymatic assay

Test compound CAGE_1:2_ (87.07% w/v) was diluted to 0.99 mM and control compound aprotinin was prepared at 6 μM in di‐water. A twofold serial dilution was then performed in di‐water to generate 10 concentrations for CAGE_1:2_ (87.07% w/v) and 8 concentrations for aprotinin which were ×2 of the final concentrations in the reaction. Recombinant human KLK5 was purchased from Speed Biosystems LLC (Catalog No. YS1485, Lot No. 0330517, 17.2 μM). The substrate S‐2288 was purchased from Diapharma Group, Inc (Catalog No. S820852, Lot No. N0889942).

The assay was carried out in 384‐well clear bottom plate coated with 0.1% Tween 20 at 4°C overnight and washed twice with di‐water before usage. Then, 50 μl 2X compound in water was mixed with 25 μl 4X hKLK5 enzyme (120 nM) prepared in 2X reaction buffer (100 mM Tris, pH 8.0, 200 mM NaCl, 0.2% PEG, 0.01% Tween 20, 2 mM EDTA). The enzymatic reaction was initiated by the addition of 25 μl 4X substrate S‐2288 (2 mM) in 2X reaction buffer to all wells. OD 405 nM was measured every 30 s by SpetraMax 384 Plus at 37°C for 1 h.

### 4.5 Human cadaver skin permeation evaluation

Dermatomed full thickness human cadaver skin samples, 250–350 μm, were mounted onto static Franz cells. The cells had a contact area of 0.7 cm^2^ and a receptor volume of 4 ml. Phosphate buffered saline, pH 7.4 was used as the receptor medium. CGB400 gel was applied to the SC side of the skin samples at a dose of ~15 μl/cm^2^ (~15 mg/cm^2^). The cells were kept in a chamber at 37°C for a period of 24 h. Stirrers in the receptor chamber ensured that the receptor media was well mixed during this time. The receptor media was sampled at 4, 7, and 24 h to quantify the amount of choline and geranic acid permeation. After 24 h, the skin surface was washed before removing the SC by tape stripping. The SC was stripped from the epidermis using an adhesive tape up to 10 layers (SC1, SC2‐5, SC6‐10). Following SC removal, the epidermis was separated from the dermis using a surgical sterile scalpel and the dermis was cut into pieces. Each layer was collected separately in glass vials containing 2 ml of 50:50 water:methanol mixture and was left to shake overnight to extract the choline and geranic acid. The amount of choline and geranic acid was measured using LC–MS/MS in the wash, epidermis, dermis, and the receptor medium.

### 4.6 Forced degradation and stability validation studies

The content of choline and geranic acid in CGB400 gel following stability and forced degradation studies were analyzed via matrix‐matched standard method using Agilent technologies 1100 and 1200 Series HPLC. System suitability, standard and method precision, linearity, accuracy/recovery and specificity were successfully established. Specificity by forced degradation was performed by stressing samples of the vehicle and CGB400 topical gel formulations. Samples were stressed with heat at 80°C oven and UV‐A light for 5 days. The heat and UV‐A stressed vehicles contained no peaks that interfered with the determination of choline. Specificity was demonstrated by comparison of the chromatographic profiles of active and stressed vehicle samples to demonstrate that no vehicle‐related degradation products elute in the retention window of choline. The loss of analyte was evaluated by the decrease in relative peak area of choline after the stress exposure. The purity factor was then calculated for the heat‐ and UV‐A‐stressed CGB400 gel. An overall purity factor of 999.85 and 999.87 for the choline peak was observed in this sample, respectively, indicating the absence of coeluting compounds with dissimilar spectra from choline.

Similarly, for geranic acid, samples were stressed with heat at 80°C oven for 5 days, UVA light for 3 days, 0.1 M hydrochloric acid for 24 h, 0.1 M sodium hydroxide base for 24 h, and 3% hydrogen peroxide for 24 h. No peaks appeared in the chromatographic profiles of UV‐, acid‐, base‐, and peroxide‐stressed samples other than heat‐stressed samples, where few new peaks appeared. Specificity was demonstrated by comparison of the chromatographic profiles of active and stressed vehicle samples to demonstrate that no vehicle related degradation products elute in the retention window of geranic acid isomers. In addition, the geranic acid peaks in the stressed active samples were evaluated by peak purity. Peak purity was evaluated from 200 to 300 nm for choline and 239 to 279 nm for geranic acid, bracketing each side of the 208 and 254 nm detection wavelength, respectively. Spectra throughout the peak were evaluated at a similarity threshold of 999 to assure that no impurity(s) with dissimilar spectra greater than 0.1% of the analytical concentration were present in the geranic acid peaks. A numerical value for this uniformity was calculated as the peak purity factor. A value of 1000 indicates no variation in the spectra for the peak. The purity factors were calculated for each geranic acid isomer in the heat, UV, acid, base, and peroxide‐stressed 40% CG‐101 gel sample.

Evaluation of the spectral data collected from the photodiode array detector was performed to determine the purity of the choline and geranic acid isomer peaks. The spectra from 200 to 300 nm for choline and 239 to 279 nm for geranic acid, at five time points (peak maximum, two points before the maximum and two points on the peak tail) were compared to each other.

For solution stability, standards and samples were prepared and analyzed on the day of preparation. For choline, the solutions were stored in sealed vials at room temperature, then reanalyzed at 72 h against freshly prepared standards. For geranic acid, the solutions were stored in sealed flasks at 5°C and room temperature, then reanalyzed after 48 h against freshly prepared standards. The validation data showed that standards and samples had stable choline levels after being stored at room temperature at least 72 h after preparation. Similarly, standards and samples for geranic acid were stable for at least 2 days after preparation when stored under refrigerated conditions.

### 4.7 Determination of dermal toxicity and TK profile in minipigs

For GLP studies, the test and control/vehicle items were administered to groups of minipigs daily by nonoccluded dermal application for 13 weeks or 91 consecutive days. The volume of test or control/vehicle items (1 ml) was applied to the dermal test site and spread uniformly over the surface of the skin, using a gloved finger but ensuring that excessive residue did not remain on the glove. Gloves were changed between each group of animals. The same dermal test site was used for all doses. Assessment of toxicity was based on mortality, clinical observations, body weight, food consumption, dermal changes, ophthalmology, electrocardiography, and clinical and anatomic pathology.

During the GLP dermal toxicity study in Gottingen minipigs, a series of six blood samples (approximately 0.5 ml each) was collected by venipuncture from each animal on Days 1 and 91 of the treatment period at the following timepoints: Predose, 1, 2, 4, 8, and 24 h after treatment. The samples were collected into tubes containing K_2_EDTA as an anticoagulant. Tubes were placed on wet ice pending processing. Following collection, the samples were centrifuged (1000*g* for 10 min at approximately 4°C) and the resulting plasma was recovered, divided into two aliquots (Sets 1 and 2) of approximate equal volume in appropriately labeled tubes and placed on dry ice pending storage in a freezer (≤−60°C). Blood samples were analyzed at a GLP bioanalytical lab in CA.

Following dosing, main animals were euthanized and subjected to a necropsy examination on Day 92. Recovery animals were observed for 14 days and then euthanized and subjected to a necropsy examination on Day 105. Animals from each group were assigned to different replicates for logistical reasons.

### 4.8 Phase Ib cosmetic study design

The goal of this study was to evaluate the ability of CGB400 in reducing facial redness, bumps/blemishes (inflammatory lesions such as papules and pustules) and determine its safety and tolerability. A multicenter, open‐label, single‐group POC study using CGB400 Gel to reduce facial redness, bumps, and blemishes was conducted. Twenty‐five subjects with redness, but without bumps/blemishes, received study treatment for 4 weeks and attended a total of five study visits (i.e., BL, W1, W2, W4, and posttreatment follow‐up at W5). Twenty‐seven subjects with redness and bumps/blemishes received study treatment for 12 weeks and attended a total of six study visits (i.e., BL, W2, W4, W8, W12, and posttreatment follow‐up at W13. CGB400 gel was applied topically to the face twice per day, once in the morning after a shower or washing the face, and once in the evening after washing the face. It was a POC study and a formal sample size justification was not provided. It was the opinion of the sponsor that 40–50 subjects would be sufficient to achieve study objectives.

Diagnosis for inclusion included:

- Subjects with facial redness and bumps/blemishes:
  - Facial redness associated with rosacea.
  - Facial redness (IGAR) score of 2 or 3 (i.e., mild or moderate).
  - IGA score of 2 or 3 (i.e., mild or moderate).
- Subjects with facial redness and without bumps/blemishes:
  - Facial redness associated with rosacea.
  - Facial redness (IGAR) score of 2 or 3 (i.e., mild or moderate).
- Effectiveness evaluations were based on:
  - IGA
  - IGAR
  - Bumps/blemishes count
  - Subject or PGA
- Safety evaluations were based on:
  - Adverse events

Statistical methods:

Analyses for effectiveness and safety were conducted on a modified intent‐to‐treat (mITT) basis. The mITT population consisted of all subjects who received at least one application of CGB400 gel and provided at least one postbaseline evaluation.

All statistical processing was performed using SAS version 9.4. For categorical parameters, the number and percentage of subjects/observations in each category were presented. The denominator was based on the number of subjects/observations appropriate for the purpose of analysis. For continuous parameters, descriptive statistics included n (number of subjects or observations), mean, SD, median, and range. Then, 95% confidence intervals were provided for all efficacy outcomes.

## CONFLICT OF INTERESTS

Patent application filed by CAGE Bio, Inc. A. M., M. S., and N. J. are employees and shareholders of CAGE Bio. J. K. and S. D. are shareholders and consultants to CAGE Bio. S. M. is a shareholder and board member/consultant to Liquideon, CAGE Bio, and i2O Therapeutics.

## AUTHOR CONTRIBUTIONS

**Justin Ko** was the safety officer and medical advisor for the design and conduct of the phase Ib study. **Sunil Dhawan** was the principal investigator for the phase Ib study. **Marina Shevachman** was involved in characterization, formulation development, and data analysis. **Nitin Joshi** was involved in characterization, formulation development, design of the GLP study, and design/conduct of the phase Ib study; and data analysis. **Samir Mitragotri** was involved in characterization, design of phase Ib study, and data analysis. **Abhirup Mandal** was involved in data summary and writing of the manuscript.

5

### PEER REVIEW

The peer review history for this article is available at https://publons.com/publon/10.1002/btm2.10191.

## Supporting information


**Figure S1** CAGE1:2 characterization, DSC, TGA and GVS analyses.
**Figure S2**. CAGE1:2 characterization, DSC thermal analysis.
**Figure S3**. CAGE1:2 characterization, FTIR analysis.
**Figure S4**. CAGE characterization, NMR and HPLC analyses.
**Figure S5**. Antimicrobial/anti‐inflammatory effects of CGB400.
**Figure S6**. TK profiling in Göttingen Minipigs Plasma on Day 1 and 91.
**Figure S7**. Effectiveness evaluations based on PGA.
**Figure S8**. Effectiveness evaluations based on IGA and IGAR.
**Table S1**. Antibacterial activity of CAGE1:2 and pure individual components against *Propionibacterium acnes*.
**Table S2**. Safety Data: Adverse event profile in Subjects WITH (n = 27) and WITHOUT (n = 25) Bumps/Blemishes.

## Data Availability

Any data associated with this study are available from the authors upon reasonable request.
