# Supplementary material for: Clinical translation of choline and geranic acid deep eutectic solvent
Source: Bioeng Transl Med. 2020 Oct 31;6(2):e10191. doi: 10.1002/btm2.10191 (PMC8126811; doi:10.1002/btm2.10191)
Supplement: Supplementary file 1 — Figure S1 CAGE1:2 characterization, DSC, TGA and GVS analyses. Figure S2. CAGE1:2 characterization, DSC thermal analysis. Figure S3. CAGE1:2 characterization, FTIR analysis. Figure S4. CAGE characterization, NMR and HPLC analyses. Figure S5. Antimicrobial/anti‐inflammatory effects of CGB400. Figure S6. TK profiling in Göttingen Minipigs Plasma on Day 1 and 91. Figure S7. Effectiveness evaluations based on PGA. Figure S8. Effectiveness evaluations based on IGA and IGAR. Table S1. Antibacterial activity of CAGE1:2 and pure individual components against Propionibacterium acnes. Table S2. Safety Data: Adverse event profile in Subjects WITH (n = 27) and WITHOUT (n = 25) Bumps/Blemishes. [file BTM2-6-e10191-s001.docx]

Title

- Clinical Translation of Choline and Geranic Acid Deep Eutectic Solvent

**Authors**

Justin Ko^1^, Abhirup Mandal^2^, Sunil Dhawan^3^, Marina Shevachman^2^, Samir Mitragotri^4,5^, Nitin Joshi^2^*

**Affiliations**

^1^Department of Dermatology, Stanford University School of Medicine, Redwood City, California.

^2^CAGE Bio, Inc. 733 Industrial Road San Carlos, CA 94070 and 181 Grand Avenue, Suite 225 Southlake, TX 76092, USA

^3^Center for Dermatology Clinical Research, Inc., Fremont, California

^4^John A. Paulson School of Engineering and Applied Sciences, Harvard University, Cambridge, MA 02138, USA.

^5^Wyss Institute of Biologically Inspired Engineering at Harvard University, Boston, MA 02115, USA

Supplementary Materials

**Materials and Methods**

**Differential Scanning Calorimeter (DSC).** Empty DSC pan and lid (hermetic Tzero) on the analytical balance were weighed. Weights were recorded and tared. Using a spatula, approximately 25 mg (viscous liquid) was transferred into the pan. The open DSC pan was then transferred to the TGA and dried for 3 hours at 60 °C (N2 flow was set at 100 mL/min for the sample). After drying, the sample was hermetically sealed using the crimper and hermetic die set. The sealed sample pan was weighed again on the analytical balance and the fill weight was calculated by subtracting the DSC pan and lid weights. The pan weights and fill weights were entered into the sample parameter section of the Q-Series control program for the Q2000 DSC. The N2 purge flow rate was set to 50 mL/min. One cool/heat run between -85 °C and 20 °C was performed using a heating ramp of 3 °C/min. Modulation was set at +/- 1.00°C every 60 seconds. A second run was performed between ca. -88 °C and 30 °C using a heating ramp of 1.00 °C/min. Modulation was set at +/- 1.00°C every 60 seconds.

**Thermogravimetric analysis (TGA).** The sample was prepared by weighing approximately 16 mg of material into a TA Pt pan. Samples were heated in the Hi-Res mode as per the method below. Nitrogen purge was set at 40 ml/min for the balance and 60 ml/min for the furnace. Data were analyzed using the TA Universal Analysis 2000 software (version 4.7B).

**Broth microdilution assay.** Serial dilutions were made in Brucella broth + 5% lysed horse blood at concentrations ranging from 6.25% liquid (1:8 dilution) – 0.0003906% liquid (1: 262,144 dilution). The liquid/broth dilutions were dispensed (100 μL/well) into 96 well microtiter trays. A positive control well was filled with 100 μL of broth only. Bacterial suspension was made in sterile pre-reduced Brucella broth, to a density equivalent to that of a 0.5 McFarland standard (108CFU/mL). Inocula were prepared from blood agar plates incubated for 48 hours. Each bacterial suspension was transferred to 10 mLs of suitable media as follows: 500 μL of P. acnes (C. acnes) was transferred to Brucella + 5% LHB. Each of the final bacterial suspensions was poured into a sterile trough and 10 μL was dispensed into each well of the microtiter tray. Colony counts were determined from the positive growth well by removing 20 μL and diluting in 10 mLs of sterile normal saline. The tube was inverted several times to mix and a 100 μL aliquot was used to inoculate a blood agar plate (BAP). The colony count plates, and MIC trays were incubated under specified conditions and growth observed after 48 hours. MICs were read as the lowest concentration (% liquid) that inhibited growth of the test organism (based on turbidity). For Geranic acid, the highest 3 concentrations were subcultured to solid media to validate the turbidity. Colonies were counted on each colony count plate (100 colonies were equivalent to 5 x 105 CFU/mL, the desired bacterial concentration).

**Suitability of AET by Plate Count Method.** The dilutions of each challenge microorganism suspensions were prepared such that the resulting level was approximately 10^5^ CFU per mL. The 1:10, 1:100 and 1:1000 dilution of the CGB400 placebo gel were prepared in TSB-M. A 1:10 dilution of the CGB400 placebo gel was prepared by adding 10g of the CGB400 placebo gel to 90 mL of TSB-M. This dilution scheme was continued to achieve 1:100 and 1:1000 dilution levels.

An appropriate number of challenge organisms was added to each tube of the diluted test article and mixed well. Two empty petri dishes were labelled for each challenge organism and 1mL of the inoculated CGB400 placebo gel dilution was plated into each dish to yield not more than 250 CFU/plate for P. aeruginosa, S. aureus, E. coli and C. albicans or not more than 80 CFU for A. brasiliensis. The volume of the suspension of the inoculum did not exceed 1% of the volume of diluted CGB400 placebo gel. In parallel, two control plates containing diluent without the CGB400 placebo gel were prepared for each organism. These served as positive controls. To show that the neutralizer is effective in inhibiting the antimicrobial properties of the CGB400 placebo gel (neutralizer efficacy) without impairing the recovery of viable microorganisms (neutralizer toxicity) a second set of positive controls using saline was prepared for each organism by inoculating the same volume of suspension dilution. Immediately after inoculation, 15-20 mL of agar appropriate for each challenge organism (TSA for bacteria, SDA for yeast and mold) was added to each dish. The plates were allowed to solidify before placing into the incubators. The TSA plates were incubated for not more than three days at 30-35°C and SDA plates were incubated for not more than three days at 20-25°C. At the conclusion of the incubation period, the number of colonies present on each plate was counted and the average CFU for the two plates was calculated.

**Method development and validation for choline in CGB400 gel.** Calibration standards were consecutively injected six times at the beginning of each analysis. Standard precision was evaluated by calculating the relative standard deviation (RSD) of the choline peak area for the first six standard injections. The precision for choline peak areas in the first six injections ranged from 0.0% to 0.2%. The acceptance criterion of not more than 5.0% RSD, specified in the USP Choline Chloride monograph, will be kept. A duplicate check standard was prepared along with the calibration standard on each day of analysis. An injection of the check standard was performed, and the response factor of the check standard injection was compared to the mean response factor of the first six injections of the calibration standard. The choline response factor of the check standard agreed with the calibration standard response factor within the 95.0 to 105.0% acceptance criterion of the method. A blank preparation was injected as part of system suitability. The chromatograms for the blank were evaluated and no peaks larger than 0.5% of the mean area of first six injections of the calibration standard were observed in the retention window of choline. Peak shape was assessed for the choline peak in the last of the six injections of calibration standard. Peak shape attributes collected were peak tailing and theoretical plates. Based on the peak tailing results, an acceptance criterion of ≤ 2.0 will be set. Based on theoretical plates results, an acceptance criterion of ≥ 5,000 will be set. The choline signal-to-noise ratio was assessed for the sensitivity solution. For all analyses the S/N was ≥ 10.

**Method development and validation for geranic acid in CGB400 gel.** Calibration standard was consecutively injected five times at the beginning of analysis for system precision check. Standard precision was evaluated by calculating the relative standard deviation (RSD) of the geranic acid peak areas for the first five standard injections. The precision for geranic acid peak areas in the first five injections met the acceptance criterion of not more than 2.0% RSD on each day of analysis. A three-point calibration curve is obtained by plotting the total geranic acid peak area of single injections for 0.75, 0.5 and 0.25 mg/mL calibration standard solutions on each day of analysis. The geranic acid calibration curve correlation coefficient was 1.000 on each day of analysis, meeting the acceptance criterion of not less than 0.997. A blank preparation was injected as a part of system suitability. The chromatograms for the blank were evaluated and no peaks larger than 0.5% of the mean area of first 5 injections of the calibration standard were observed in the retention window of geranic acid.

Peak shape was assessed for the Geranic Acid 2 peak in the last injection of the 0.5 mg/mL calibration standard for system precision check. Peak shape attributes collected were peak tailing, theoretical plates, as well as the resolution between Geranic Acids 1 and 2. In all cases, the peak tailing was 1.1 and the theoretical plates greater than 250,000 were obtained for Geranic Acid 2 isomer. Resolution between the Geranic Acid 1 peak and the Geranic Acid 2 peak ranged from 1.51 to 2.09. Since the loss of peak efficiency and peak symmetry will directly result in loss of resolution, monitoring resolution will be a sufficient suitability test of peak shape. An acceptance criterion of no less than (NLT) 1.5 will be set for resolution. The signal-to-noise (S/N) ratio was assessed for the sensitivity solution. The S/N for all analyses was greater than 10.

To demonstrate the consistency of the instrument throughout the run, bracketing standards were injected after a maximum of six sample injections. The response factor of each bracketing standard injection was compared to the mean response factor of the first five injections of the 0.5 mg/mL calibration standard. The geranic acid response factor of each bracketing standard ranged from 97.0% to 100.1% with the calibration standard response factor. Therefore, an acceptance criterion of 97.0% to 103.0% will be proposed for the method.


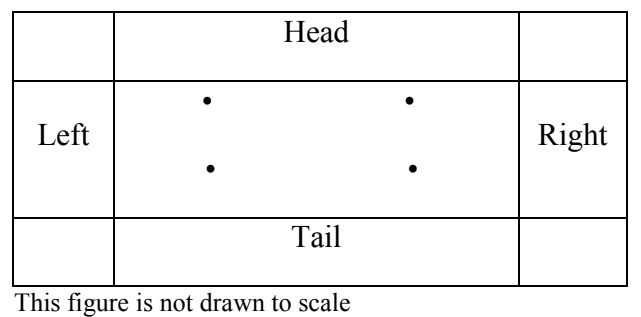
**Test system preparation and dosing in GLP minipig study.** During the pre-treatment period (between Days -5 and -2), an area on the upper dorsum (across the midline) of each minipig was clipped free of hair. This area was limited in size from approximately the rump to the lower neck region and halfway down the flanks. The dermal test site was cleared of hair as often as necessary and care was taken to not damage the skin. Any damage caused mechanically was noted in the raw data. The dermal test site was defined by dots in each of the four corners using an indelible ink following the Day -1 body weight (to allow calculation of 10% Total Body Surface Area [BSA]). The area does not necessarily need to be a square; a rectangle is acceptable as long as the % BSA is adhered to.

The dermal test site (area of application) was calculated as 10% of the Total Body Surface Area of the animal (i.e. area of skin by the body weight) on Day -1 and weekly thereafter, using the following formula, which is specific to the minipig:

BSA (m2) = (BW (kg) * 1000) ^ (2/3) *k/10000

Where BSA = body surface area of the animal, BW = body weight and the value of k = 9. (Formula by Meeh). If the dermal test site size increased during the study, the anchoring point was the left corner nearest to the head.

**Washing of the dermal site in minipigs.** On all dosing occasions, with the exception of Day 1, the dermal test site was wiped clean using water moistened gauze prior to dosing to remove any residue of formulation from the skin. Washing was performed using warm reverse osmosis water. Unscented soap was used if deemed necessary. The dermal test site was dried with soft tissue, using gentle pressure.

**Toxicokinetic studies**. A series of 6 blood samples (approximately 0.5 mL each) was collected by venipuncture from each animal on Days 1 and 91 of the treatment periods at the following timepoints: Pre-dose, 1, 2, 4, 8, and 24 hours after treatment. The samples were collected into tubes containing an appropriate anticoagulant (to be added via amendment). Tubes were placed on wet ice pending processing. Following collection, the samples were centrifuged (1000 g (approximately 2500 rpm) for 10 minutes at approximately 4°C) and the resulting plasma was recovered, divided into two aliquots (sets 1 and 2) of approximate equal volume in appropriately labelled tubes and placed on dry ice pending storage in a freezer (≤ -60°C).

**Bioanalytical method for determining choline and geranic acid in minipig plasma samples**. Concentrations of Geranic Acid and Choline were determined in 373 Minipig plasma samples by an LC/MS/MS method. In the method, 10 µL of standard spiking solutions of Geranic acid of appropriate concentrations were added to individual 190 µL aliquots of blank minipig plasma for preparing Geranic Acid calibration standards and QCs. 120 µL of MeOH was added to 40 µL of plasma and centrifuged at 4000 rpm for 10 minutes. For choline, 64 μL of internal standard solution (choline-d9) was added to 16 μL H_2_O. All the calibration standards and QCs were freshly made from stock solution stored at -20 °C freezer. The Shimadzu Prominence HPLC system (PMO-0198, 0163, 0156 and 0201) combined with AB Sciex API 5000 (PMO-0193) was used to perform the LC/MS/MS analysis of Choline, and the Shimadzu Prominence HPLC system (PMO-0197, 0162, 0188 and 0202) combined with AB Sciex API 5000 (PMO-0226) was used to perform the LC/MS/MS analysis of Geranic Acid. With a 2 µL and 10 µL of sample injection, Choline and Geranic Acid, respectively, were separated from interfering substances and subsequently eluted from the HPLC column for mass quantification. An MRM mode of mass spectrometer analysis was used to detect and quantify Geranic Acid and Choline. Data were processed and final concentrations of samples were calculated by an automated data acquisition and process system - Analyst 1.4.2 (Applied Biosystems, Foster City, CA).

Supplementary Materials

**
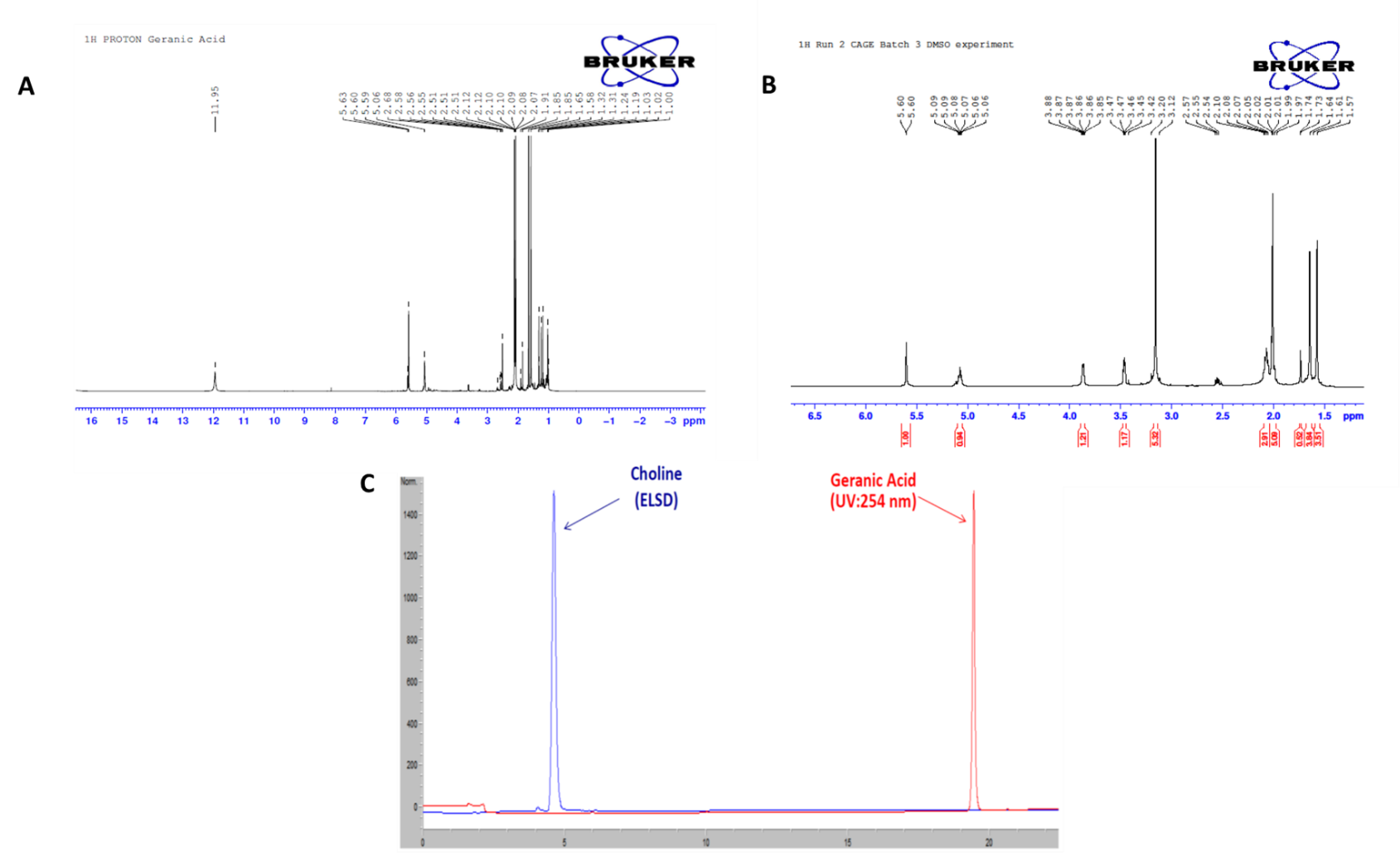
**

**Fig S1.** **CAGE characterization, NMR and HPLC analyses.** (A, B) NMR spectra of geranic acid, and CAGE_1:2_ respectively; (C) HPLC method development for choline and geranic acid.


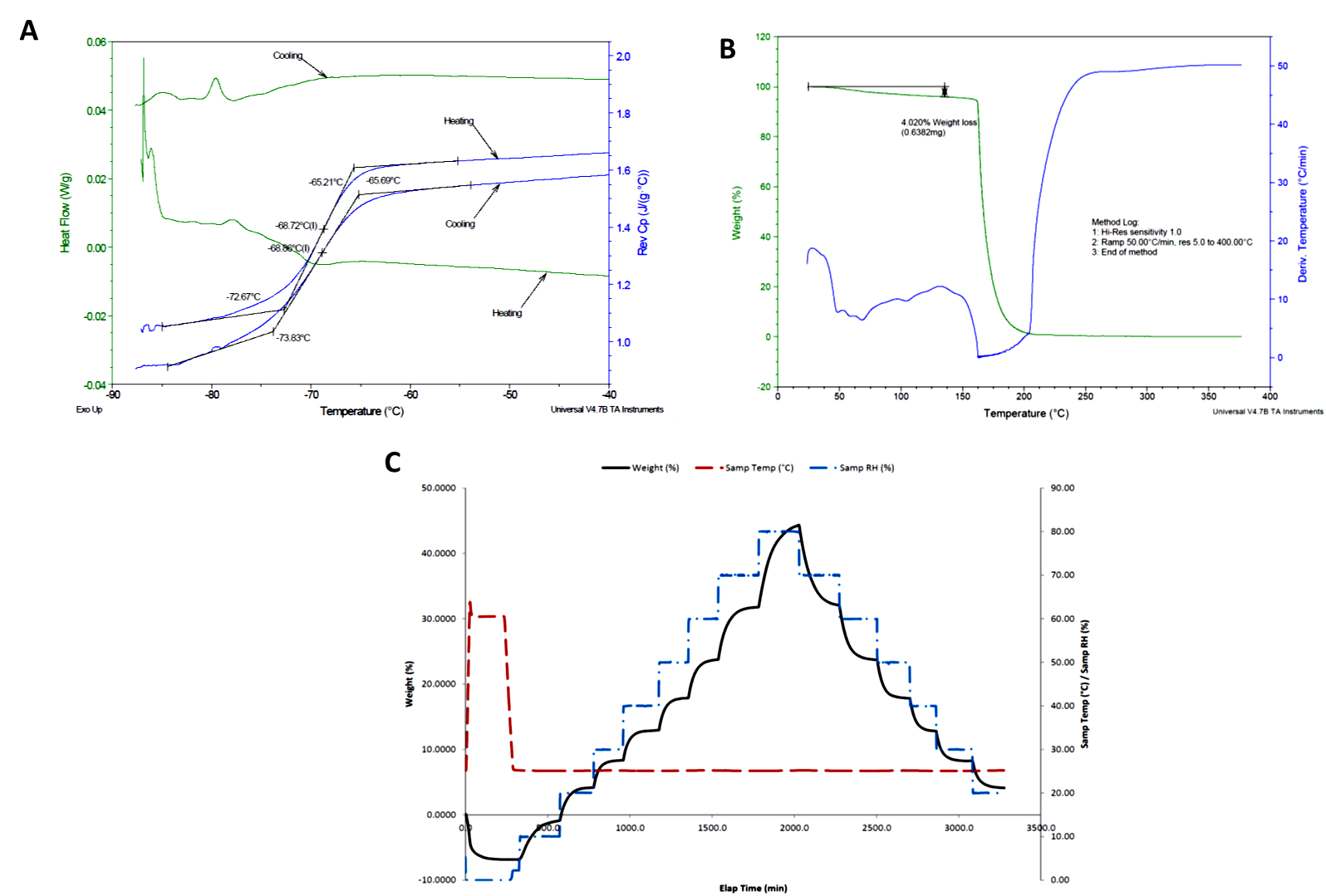


**Fig. S2.** **CAGE_1:2_ characterization, DSC, TGA and GVS analyses.** (A) DSC spectra modulated thermal analysis of CGB400 gel; (B) TGA analysis of CGB400 gel measuring %wet weight of water loss; (C) GVS spectra indicating water uptake with respect to dry weight.

**
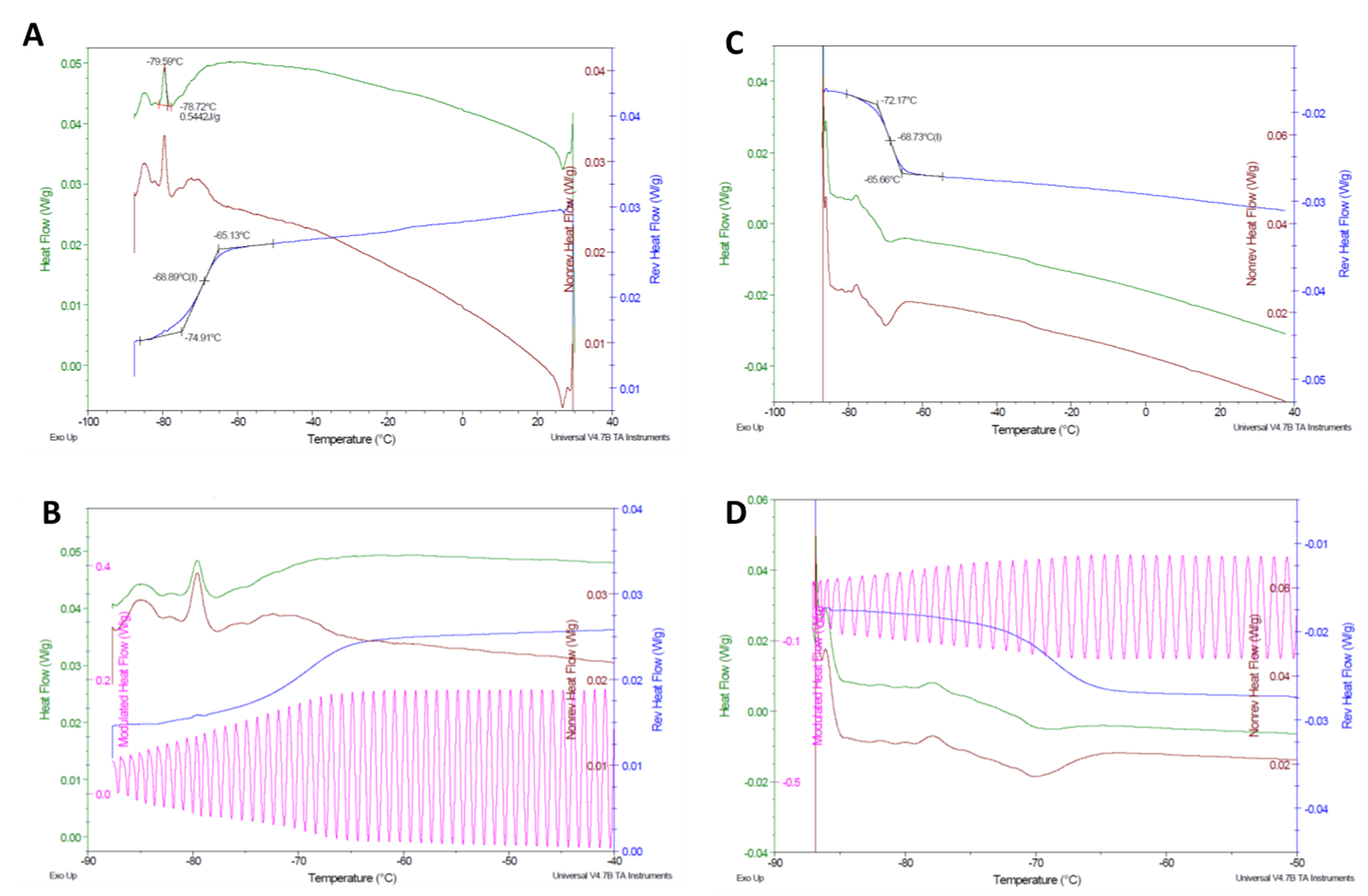
**

**Fig. S3.** **CAGE_1:2_ characterization, DSC thermal analysis.** (A) Cooling cycle. Glass transition marked in Rev Heat Flow (~-69C). Small exotherm in total heat flow (~-80C); (B) Cooling cycle illustrating modulated heat flow, expanded at low temperature. Some signal distortion < ~-80 °C. Exotherm in nonreversing heat flow ca. -80C; (C) Heating cycle. Glass transition marked in Rev Heat Flow (~-69C). Slight endotherm near glass transition. Slight endotherm in total heat flow (~-70C) and more apparent in nonreversing heat flow near glass transition, possibly enthalpic relaxation; (D) Heating cycle illustrating modulated heat flow, expanded at low temperature. Change in modulated heat flow consistent with glass transition. Some signal distortion < ~-80 °C.

**
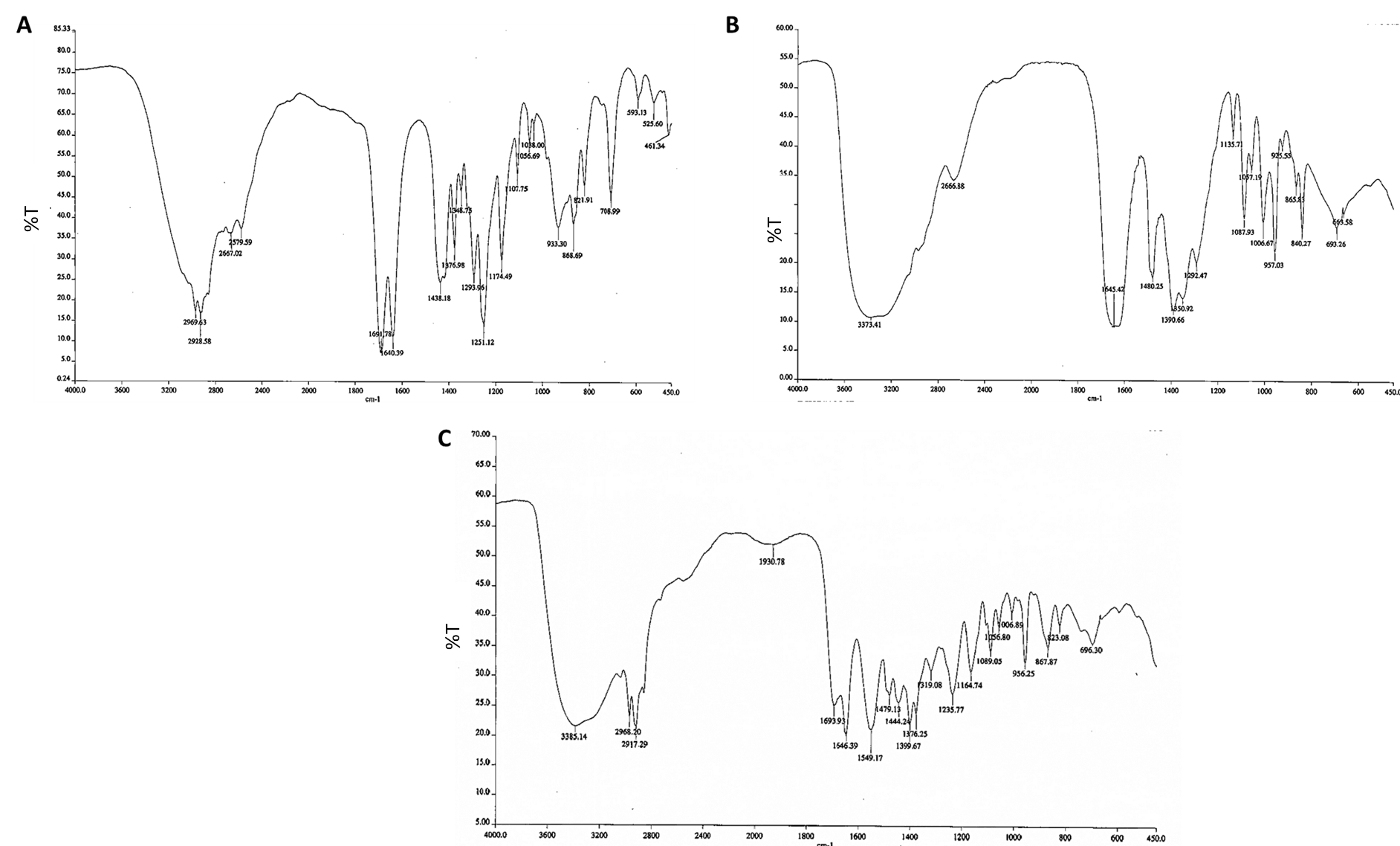
**

**Fig S4.** **CAGE_1:2_ characterization, FTIR analysis.** FTIR spectra of choline (A), geranic acid (B), and CAGE_1:2_ (C).

**
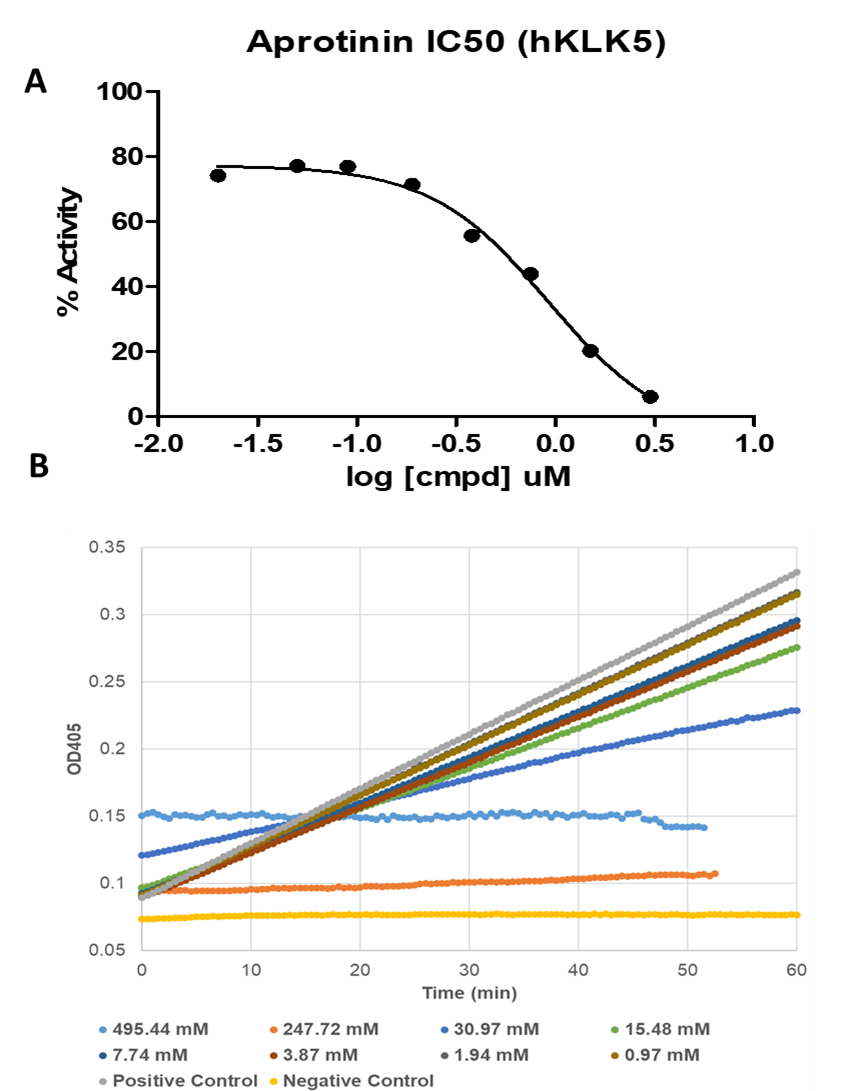
**

**Fig S5.** **Antimicrobial/anti-inflammatory effects of CGB400.** (A) IC50 curve of Aprotinin (positive control); (B) OD 405 nm Reading of hKLK5 Inhibition by CAGE_1:2_

**
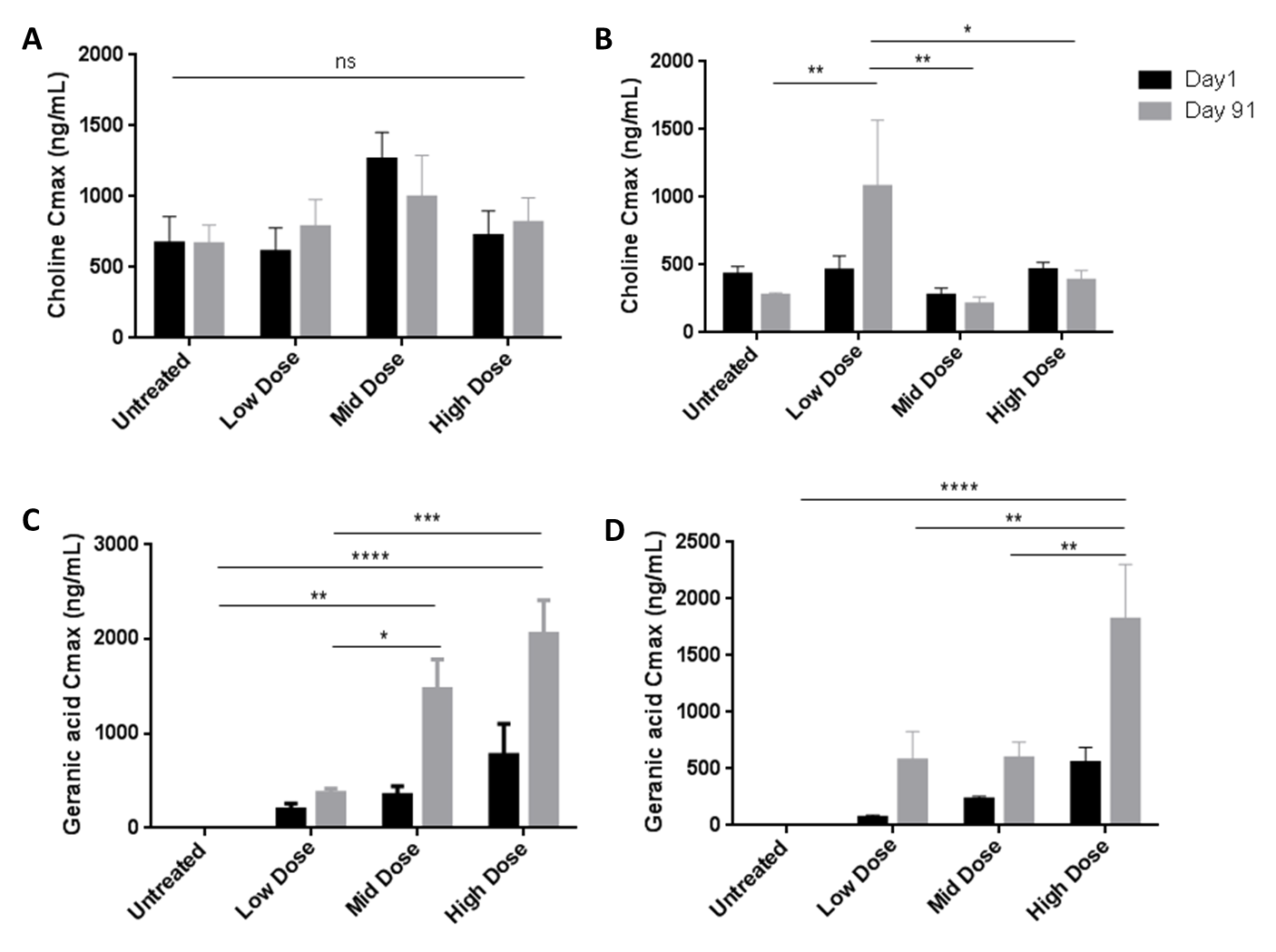
**

**Fig S6. TK profiling in Göttingen Minipigs Plasma on Day 1 and 91.** Maximum mean plasma concentrations (Cmax) of choline in (A) males; (B) females and geranic acid in (C) males; (D) females. For choline, there was a lack of dose response, however for geranic acid, Cmax increased dose dependently and in a more-than dose proportional manner. Data are averages ± SEM, statistics by two-way ANOVA with Tukey HSD post-test. *P<0.05, **P < 0.01, ***P<0.001, ****P < 0.0001.

**
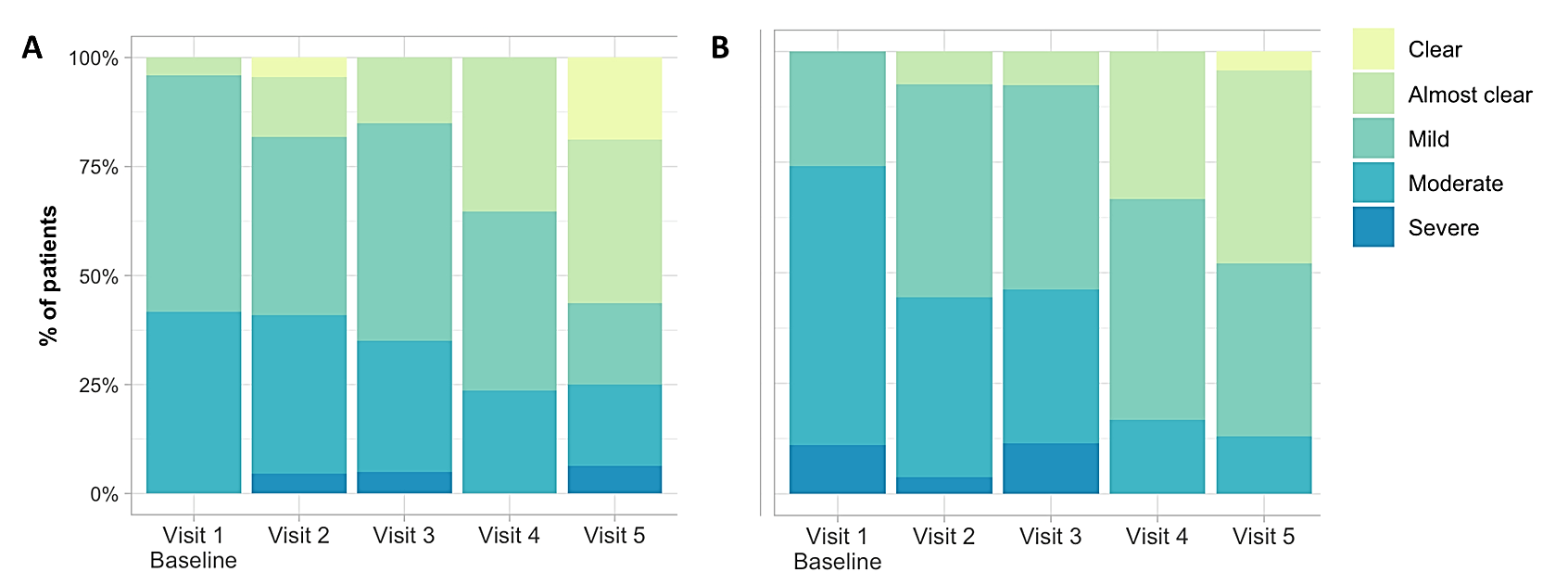
**

**Fig. S7.** **Effectiveness evaluations based on PGA.** Quantification of redness per visits based on PGA in subjects with (A) Redness only; and (B) Redness with Bumps/Blemishes.


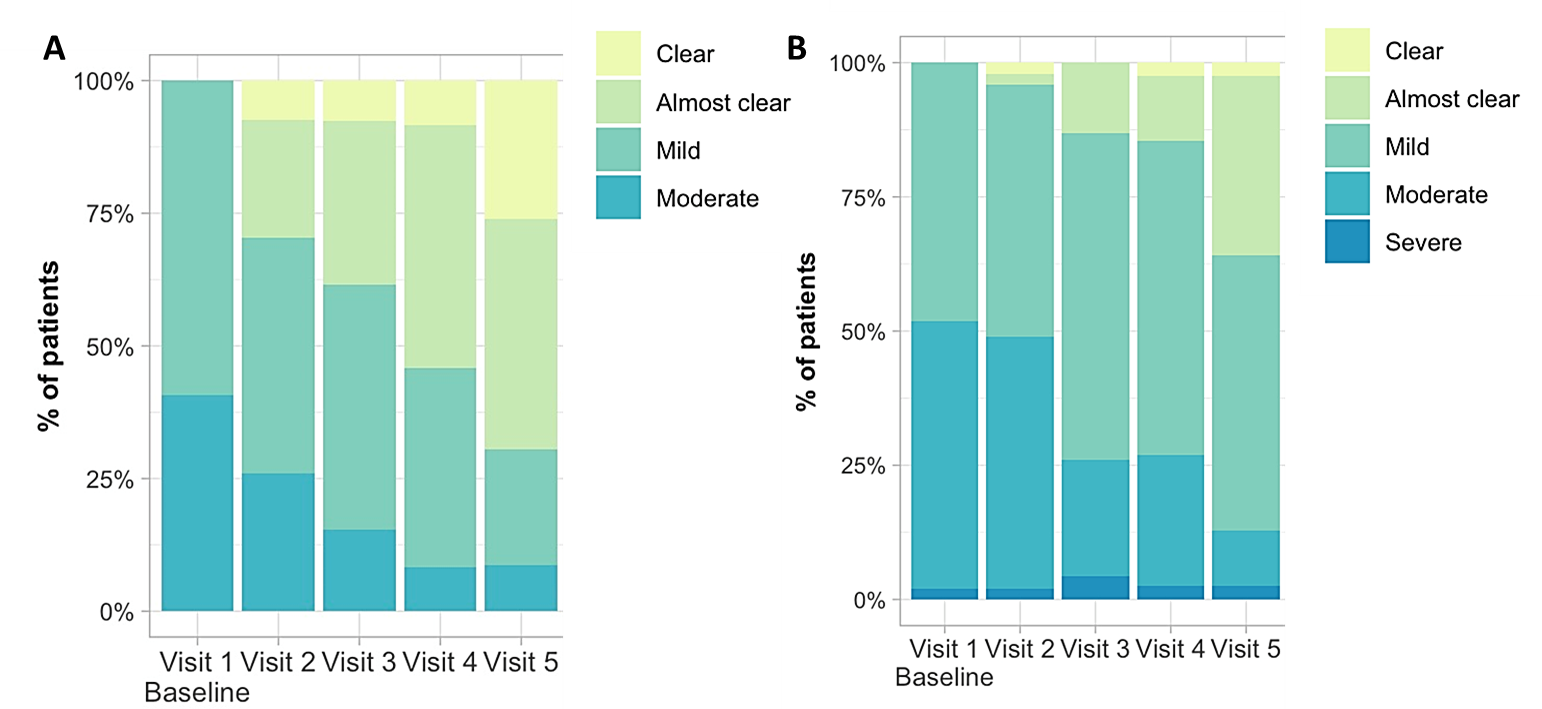


**Fig. S8.** **Effectiveness evaluations based on IGA and IGAR.** Quantification of redness per visits based on (A) IGA in subjects with Bumps/Blemishes; (B) IGAR in all subjects.

**Supplementary Tables**

PAC = *P.acnes*

*Growth in all wells (as determined by turbidity and subculture from the wells). A 1:32 was the least dilution we could start with before the compound affected the testing medium.

**Table S1.** Antibacterial activity of CAGE_1:2_ and pure individual components against *Propionibacterium acnes.*


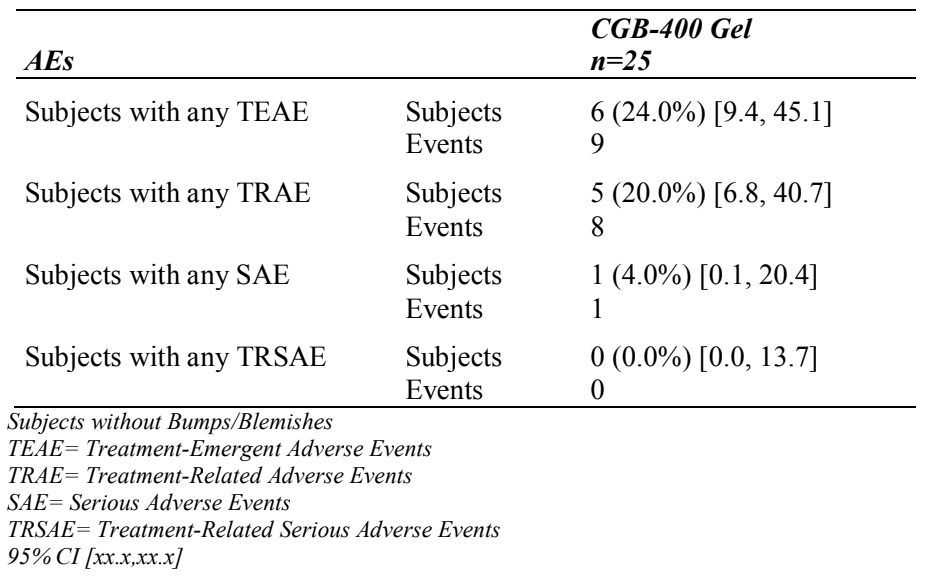

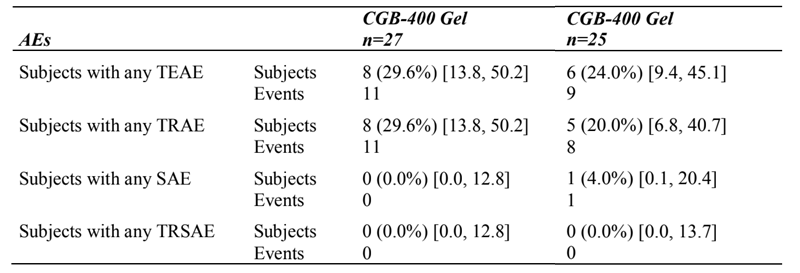


**Table S2.** Safety Data: Adverse event profile in Subjects WITH (n=27) and WITHOUT (n=25) Bumps/Blemishes.
